# Antibacterial Activity Test of Extracts and Fractions of Cassava Leaves (*Manihot esculenta* Crantz) against Clinical Isolates of *Staphylococcus epidermidis* and *Propionibacterium acnes* Causing Acne

**DOI:** 10.1155/2020/1975904

**Published:** 2020-01-27

**Authors:** Resmi Mustarichie, Sulistiyaningsih Sulistyaningsih, Dudi Runadi

**Affiliations:** ^1^Pharmaceutical Analysisi and Medicinal Chemistry Department, Faculty of Pharmacy, Farmasi Universitas Padjadjaran, Sumedang 45363, Indonesia; ^2^Biology Pharmacy Department, Faculty of Pharmacy, Universitas Padjadjaran, Sumedang 45363, Indonesia

## Abstract

This study is aimed at determining antibacterial activity from ethanol extracts and the most active fraction of cassava leaves against clinical isolates of *Staphylococcus epidermidis* and *Propionibacterium acnes*. Research carried out by the experimental method involved determination of plants, extraction with maceration method, fractionation with liquid-liquid extraction, antibacterial activity testing of extracts and fractions by agar diffusion method, determination of most active fraction from the extract, and minimum inhibitory concentration (MIC) and minimum bactericidal concentration (MBC) testing of most active fraction by microdilution method. The results showed that ethanol extracts of cassava leaves had antibacterial activity against both bacteria with the most active fraction indicated by ethyl acetate. MIC values of ethyl acetate fraction against *S. epidermidis* were in the concentration range of 2.5%–5.0% (w/v) and against *P. acnes* were in the concentration range of 1.25%–2.5% (w/v). The MBC value of ethyl acetate fraction against *S. epidermidis* was at a concentration of 5% (w/v), while *P. acnes* was at a concentration of 2.5% (w/v). From the results of this study, it can be concluded that the ethanol extract of cassava leaves (*Manihot esculenta* Crantz) has antibacterial activity against clinical isolates of *Staphylococcus epidermidis* as well as on *Propionibacterium acnes*. The fraction with the best activity from the ethanol extract of cassava leaves to the two test bacteria was shown by ethyl acetate fraction. It is suggested that cassava leaves are possible to be developed into standardized antiacne herbal.

## 1. Introduction

Acne vulgaris or acne is a chronic inflammatory disease of pilosebaceous follicles characterized by the appearance of blackheads, papules, pustules, and nodules [1]. Acne vulgaris results from a disturbance of keratinization accompanied by blockage of sebum flow produced by oil glands in the hair follicle ducts and primary acne efflorescence arising from blackheads or boils that protrude on the surface of the skin [2]. The site of the spread of the disease is limited to the pilosebaceous follicles on the face, chest, and back because the sebaceous glands in this area are very active [3, 4]. The prevalence of zits varies depending on age and sex. The incidence of acne vulgaris ranges from 85% and occurs at the age of 14–17 years in women and 16–19 years in men, with predominant lesions being blackheads and papules. Acne can occur in children aged 9 years, but the peak is in men, especially those aged 17-18 years while women aged 16-17 years. Acne vulgaris is generally more common in men than women in the age range of 15–44 years, i.e., 34% in men and 27% in women [5].

Microbial colonization in the sebaceous gland, such as Gram-positive bacteria *S. epidermidis* and *P. acnes* can potentially cause acne in certain conditions. These bacteria are not pathogenic under normal conditions, but if there are abnormal skin conditions, such as blockages in the ducts of the pilosebaceous gland secretion and increased production of sebum, then these bacteria turn into invasive [6, 7]. Invasive *S*. *epidermidis* and *P*. *acnes* can produce lipolytic enzymes that convert sebum fractions into dense periods that damage hair follicles, causing comedogenic and inflammatory effects on the skin surface [8, 9]. Until now, there have been no healing methods that completely treat acne. Although there are methods of antibacterial treatment with topical use of synthetic antibiotics such as clindamycin, tetracycline, and erythromycin [10, 11], but along with improper use of antibacterial agents (especially antibiotics) for a long time, there is an increasing development of microorganisms that cause disease, causing new types of bacteria that are resistant to antibiotics [12]. Therefore, it is necessary to look for alternative treatments, one of which is the use of herbal plants that have the potential as antibacterial.

As a tropical country, plants are one of the richest natural resources in Indonesia. Among the many types of plants that exist, cassava (*Manihot esculenta* Crantz.) is one of the plants used in everyday life. In Indonesia, cassava is a food ingredient that is widely consumed by the community and is a staple food in several regions after rice and corn. In addition to the utilization of cassava tubers as a staple food, cassava leaves are also used by ordinary people to overcome and treat ulcer disease, rheumatism, gout, diarrhea, fever, headache, night blindness, intestinal worms, and beri-beri disease [13]. Based on several scientific studies that have been carried out, cassava leaves are known to have chemical constituents including carbohydrates, calcium, phosphorus, fat, protein, vitamin A, vitamin B1, vitamin B2, vitamin C, water, substances, iron, flavonoids, saponins, and triterpenoids [14–16]. Flavonoid and saponin compounds are known to have antimicrobial and antiviral activity. Likewise, triterpenoids are known to have antiviral and antibacterial activity and can treat damage to the skin [17]. Other studies that have been conducted on cassava leaves include routine level checks, exploration of chlorophyll content as a basic food supplement, utilization in the wound healing process, anti-inflammatory, antioxidants, anticancer, and larvicide of mosquito *Aedes aegepti*; however, scientific studies so far relating antibacterial activity to ethanol extract of cassava leaves *M. esculenta* are still limited [18–20].

On the basis that so far there are no articles that discuss the antibacterial activity of cassava leaves extracted against *S. epidermidis* and *P. acnes*; this study reports scientific information about the benefits of extracts and fractions of cassava leaves as substances that have the potential to have antibacterial activity against *S epidermidis* and *P. acnes* which cause acne.

## 2. Materials and Methods

### 2.1. Materials

Cassava plants were obtained from Lembang, West Java and determined in the taxonomy laboratory, Department of Biology, FMIPA-Universitas Padjadjaran.

### 2.2. Chemicals

Fuxine water, Dragendorff reagent, Lieberman–Buchard reagent, and Mayer reagent were used. If not stated otherwise, all chemicals were of analytical grades.

### 2.3. Medium for Bacterial Growth

Mueller Hinton Agar (Oxoid, Basingstoke, UK) and Mueller Hinton Broth (Oxoid, Basingstoke, UK) were used.

### 2.4. Microbial Test

*S. epidermidis* and *P. acnes* clinical isolates were used.

#### 2.4.1. Preparation and Sterilization of Medium

38 g of Mueller Hinton Agar (MHA) was dissolved in 1 L of distilled water, 21 g of Mueller Hinton Broth (MHB) was dissolved in 1 L of water, and then physiological NaCl was prepared for the bacterial suspension, thus making the medium. Then, the medium was sterilized in an autoclave at 121°C for ±15 minutes.

#### 2.4.2. Tools

Tools such as a thin-layer chromatography set (Camag), toluene distillation equipment (Barnstead), incubator (Sakura IF−4), 254 nm and 366 nm UV lamps (Camag), refrigerator (Electrolux), 96-well microplate (Iwaki), light microscope (Nikon), analytic balance (Mettler Toledo), and glass which were commonly used in Laboratory of Pharmaceutical Microbiology, Universitas Padjadjaran.

#### 2.4.3. Methods

The study began with material collection and plant determination by selecting cassava leaves, which were not affected by pests, collected, sorted, and dried until it beccome a sample [21, 22].

#### 2.4.4. Extraction

Cassava leaves were macerated using 96% ethanol. The selection of this method was done to prevent the occurrence of damage to the thermolabile chemical compounds contained in the cassava leaves. The maceration was carried out by soaking the sample in the macerator and then leaving it for 24 hours at room temperature along with occasional stirring. The solvent replacement was carried out during 3 × 24 hours [23–25].

### 2.5. Phytochemical Screening

Phytochemical screening was carried out to detect secondary metabolites in the sample and ethanol extract of cassava leaves. Screening is carried out by the method stated in the Biological and Phytochemical Screening of Plants [26].

### 2.6. Extract Parameters

Testing the sample standard parameters included organoleptic test, water content determination, drying losses, water-soluble and ethanol extract content, total ash content, water-soluble ash content, and acid-insoluble ash content. Tests of extract parameters included organoleptic test, water content determination, drying losses, water-soluble extracts, and ethanol. The procedure was carried out according to what is stated in the attachment of Indonesian Herbal Pharmacopoeia Edition I [27].

### 2.7. Bacterial Identification Test

Test of bacterial affirmation test included observation of colony morphology, Gram staining, and biochemical tests.

### 2.8. Antibacterial Activity Test Extract

The antibacterial activity test of cassava leaf extract was carried out by the diffusion method using the perforation technique. A total of 20 *μ*L of each test bacterial suspension was put into sterile Petri dishes, and then 20 mL of sterile MHA was still added (temperature 45–50°C). The mixture is homogenized, and then allowed to solidify at room temperature. Culture media that have solidified are perforated using holes. In each of these holes, 50 *μ*L of ethanol extract of cassava leaves were added with concentrations of 30%, 20%, 10%, and 5% (b/v) using a micropipette. The test media were then incubated in an incubator at 37°C for 18–24 hours and then measured the diameter of the inhibition zone formed using a caliper.

#### 2.8.1. Fractionation

Fractionation of the extract was carried out by the liquid-liquid extraction (LLE) method using two solvents that were not mixed [28]. Thick fractions of n-hexane, ethyl acetate, and water were obtained. All fractions are observed in the organoleptic form including the color odor and then calculated the yield of each fraction by the formula:

(1) $$\begin{array}{c} \text{Fraction yield}\left(\%w/w\right)=\frac{\text{wt fraction}}{\text{s sample}}100\%. \end{array}$$

### 2.9. Fraction Antibacterial Activity

The antibacterial activity of cassava leaf fraction was carried out by the diffusion method using a perforation technique such as in the antibacterial activity of extracts. The test was carried out using the smallest concentration which still provided activity in testing the extract. The most active fraction of the ethanol extract of cassava leaves was determined by the diameter of the largest inhibition zone.

### 2.10. Determination of Minimum Growth Inhibitory Concentration (MIC) and Minimum Bactericidal Concentration (MBC)

Determination of MIC and MBC fraction of the ethanol extract of cassava leaves against *S. epidermidis* and *P. acnes* clinical isolates was carried out by the microdilution method [29]. Columns 1–12 of the microdilution plate are filled with 100 *μ*L MHB. The first column is the negative control, the second column is the negative fraction control (MHB and fraction), and the twelfth column was the positive control (MHB and test bacteria). Column 3 was filled with 100 *μ*L of the most active fraction at a concentration of 10%. The dilution process was done by piping 100 *μ*L from the 3rd column to the 4th column. This process was done repeatedly until column 11, and then 100 *μ*L of the final dilution results were removed. Therefore, the concentration of the microdilution plate (from 3rd to 11th column) would be obtained at 10%, 5%, 2.5%, 1.25%, 0.625%, 0.3125%, 0.15625%, 0.078125%, and 0.0390625 b/v. In columns 3 to 12, 10 *μ*L of the test bacteria were inserted.

### 2.11. Determination of the Most Active Thin-Layer Extract and Fraction Chromatography Profiles

Determination of the thin-layer chromatography (TLC) profile was carried out to determine the profile of the compounds contained in the extract and the most active fractions. The stationary phase used was GF 254 silica gel, and the mobile phase was n-butanol :  acetic acid : water with a ratio of 4 : 1 : 5. At the initial line (within 0.5 cm from the edge), the silica gel plate was 5 cm × 2 cm, the ethanol extract was bottled, and the most active fraction was using capillary pipes. The spots obtained were then observed in visible light, 254 nm UV light, and 366 nm UV light and the Rf value was calculated.

## 3. Results and Discussion

### 3.1. Extraction Results

Extract of cassava leaves was carried out using the maceration method by soaking 450 grams of sample using ethanol 96% solvent at room temperature (20°C–25°C). The maceration method was chosen by considering the components of secondary metabolites in sample. Extraction in this method used room temperature so that the decomposition of secondary metabolites could be avoided. 96% ethanol solvent was used as a solvent because it could extract almost all secondary metabolites present in sample. This property was due to the presence of the -OH group which was polar, while the ethyl group (CH3CH2-) was nonpolar. With short carbon chains, ethanol was semipolar which was capable of dissolving nonpolar and polar secondary metabolites. Extraction was carried out 3 times with a take-up time of 24 hours storage [23–25]. This method was chosen because it was a fast, easy method and can remove solvents properly without damaging the secondary metabolites in the extract. The evaporation results that had been obtained were extracted and then stored in the evaporator cup and reheated on a water bath at a temperature of 30°C–40°C to remove the remaining residual solvents, until the thick extract period was obtained, and the yield was calculated. Of the 450 g of the sample used, the ethanol extract was 98.43 g so that the yield of ethanol extract of cassava leaves was 21.873% (w/w). The yield value was related to the number of secondary metabolites that were successfully extracted by comparing the weight of the extract to the weight of the sample [22]. Organoleptic observation showed that the extract was blackish-brown, distinctive smelling, and had bitter taste.

### 3.2. Phytochemical Screening

Phytochemical screening aimed to determine the secondary metabolites contained in sample and ethanol extract of cassava leaves with test results which can be seen in Table 1.

Thiyagarajan and Suriyavathana reported their study on phytochemical and antimicrobial screening of *M. esculanta* Crantz varieties Mulluvadi and found their sample contained alkaloid and steroids [30]. The presence of antioxidant flavonoids and phenols was reported by Quartey et al. [31].Effect of boiled cassava leaves (M. esculenta Crantz) on total phenolic, flavonoid, and antioxidant activity was reported by Appiah et al. [32]. The difference in the results of the phytochemical screening analysis might be due to the origin of plants which were affected by soil nutrients in each region.

### 3.3. Standard Sample and Extract Parameters

The standard parameters of the sample tested included water content, water-soluble extract content, ethanol-soluble extract content, drying losses, total ash content, water-soluble ash content, and acid-insoluble ash content. As for the extract parameters, the procedure carried out was the same as the standard sample parameter, but without testing the ash content [27]. The results of testing the standard sample parameters and extracts can be seen in Table 2.

From Table 2, it could be concluded that the water content of the extract was greater than the water content of the sample but still satisfies the water content of the extract according to Soetarno and Soediro which should not be more than 10% to avoid rapid growth of fungi and molds [33]. However, the presence of water content in ethanol extract showed that the evaporation process was poor. Tests for drying losses were carried out to provide maximum limits (ranges) about the number of compounds lost in the drying process. From the data in Table 2, after testing, the shrinkage of the sample was greater than the drying loss in the extract. This was because the sample used still had water content and other compounds that could be evaporated during the testing process at a temperature of 100–105°C, resulting in a reduction in the total period of the sample after testing. Whereas in the extract, the drying shrinkage was smaller because the solvent used was mostly evaporated in the evaporation process. In testing the ash content in the sample, the principle was that the material was heated at a high temperature (600°C) where the organic compound and its derivatives decompose and evaporate until only the organic mineral elements remain so that the determination of ash content aims to describe the internal and external mineral content in the sample, starting from the initial process until the extract was formed. The levels of acid-soluble ash were examined to determine the level of the contents of calcium carbonate and alkali chloride, while the levels of acid-insoluble ash were examined to determine the level of contamination by metals and silicates. From the data in Table 2, after testing, the total ash content of the sample obtained was 13%, of which 92.31% of the ash content was water-soluble and was 7.69% insoluble in acid. The purpose of testing the standard sample parameters and extract parameters was to standardize sample and determine extract quality. If the sample or extract from the same plant was tested again but if it gave different results, it was not necessarily the result of the test was wrong because the test results were influenced by compounds or substances contained in sample or extracts, which could be seen from testing the standard parameters. For example, the determination of soluble extracts of ethanol and levels of water-soluble extracts were specific parameters and parameters of total ash content were nonspecific parameters. The parameters of soluble extracts of ethanol and the levels of water-soluble extracts were said to be specific because they showed the solubility properties of the chemical compounds contained in the sample and extracts with a search using different solvents of polarity, while the nonspecific ash content parameters because the amount of metal contamination and the content of inorganic substances could differ even though the plants were the same depending on the place and condition of the test plant when taken so that it could reduce the test results or increase the test results. This was why it was necessary to test the standard sample and extracts, such as in herbal medicine and medicinal products from natural ingredients that had been on the market.

### 3.4. Identification of Test Bacteria

Before the antibacterial activity of the extracts was tested, a test of bacterial confirmation was carried out to ensure that the test bacteria were used. The bacteria used in this study were clinical isolates of *S. epidermidis* and *P. acnes*. First, the identification of the clinical isolates of *S. epidermidis* bacteria was carried out. The first identification was carried out by growing bacteria on the media to see the colony morphology of the test bacteria. From the results of bacterial growth on agar media, the *S. epidermidis* test bacteria grew with round, opaque, yellowish-gray colonies. These results were following the colony morphology found in the literature [34]. Next, Gram identification was done to separate the types of bacteria into Gram-positive bacteria and Gram-negative bacteria. From the results of microscopic observations of light at 1000x magnification, the test bacteria used were round-shaped like grapes and purple in color. This showed that the *S. epidermidis* bacteria used were included in the group of Gram-positive bacteria. These results were as per the literature which stated that *S. epidermidis* belong to Gram-positive bacteria [34]. Then, another affirmation test was carried out, namely, the biochemical test. This test was carried out to look at the biochemical properties of the test bacteria from their metabolic results against certain reagents after incubation at 37°C for 18–24 hours. The biochemical test results gave different results for each bacterium, so it was necessary to do an affirmation test in addition to general Gram staining. The biochemical test results of *S. epidermidis* test bacteria can be seen in Table 3.

From Table 3, it could be seen that, in the biochemical TSIA and Voges–Proskauer tests, the results shown in *S. epidermidis* clinical isolates were negative whereas in the literature, the results were shown in positive. Biochemical test results were slightly different from the literature because the bacteria used were clinical isolates so they had different characteristics when compared with the literature. Langlois et al. reported that existing species and biochemical characteristics tend to vary with sample sources and geographical regions [35]. Based on the results of this bacterial affirmation test, it could be concluded that the true test bacteria used were *S. epidermidis* bacteria. Then, the identification of the second test bacteria, *P. acnes* clinical isolates, was also carried out. The first identification was carried out by growing bacteria on the media to see the colony morphology of the test bacteria. From the results of bacterial growth on agar media, *P. acnes* test bacteria grew morphologically in the form of stems, opaque, and white in turbidity. The next identification was Gram staining. From the results of microscopic observations at 1000x magnification, the rod-shaped bacteria were irregular and purple. This showed that the test bacteria used to belong to the group of Gram-positive bacteria. Microscopic results and Gram staining of test bacteria can be seen in Table 4.

From Table 4, it showed that the test results were the same as the test results found in the literature [34, 36] except in the glucose biochemical test, in which the results were shown in *P. acnes* clinical isolates were in negative, whereas in these textbooks, the results were shown in positive. However, negative glucose results from clinical isolates of *P. acnes* had also been reported by Polugari et al. [37]. As well as in our study, the use of clinically isolated *P. acnes* was not a pure *P. acnes* species that might be caused. Clinical isolate bacteria most likely had different characteristics from the original species. Based on the results of this bacterial affirmation test, it could be concluded that the actual bacteria used were *P. acnes* bacteria.

### 3.5. Antibacterial Extract Activity Test

It was found that the ethanol extract of cassava leaves at a concentration of 5% showed no activity against the bacterial isolates of clinical *S*. *epidermidis* and still showed activity in clinical isolates of *P. acnes*. The greater the concentration of the extract solution, the greater the inhibition zone of the two test bacteria.

### 3.6. Extract Fractionation

Fractionation of ethanol extract of cassava leaves was carried out through a liquid-liquid extraction process (ECC), with the principle of the partition using solvents that did not mix, which aims to separate secondary metabolites in extracts based on their level of polarity [28]. It was found to possess 3 fractions, namely, n-hexane fraction of yield of 1.302%, ethyl acetate fraction of 0.348%, and water fraction of 2.56%. The organoleptic observations of the three fractions showed that the n-hexane fraction was solid, blackish green, distinctive smelling, and had bitter taste, ethyl acetate fraction in the form of blackish-brown thick solid, distinctive smell, and had bitter taste, while the water fraction was brown thick liquid blackish, distinctive smelling, and had bitter taste.

### 3.7. Fraction Antibacterial Activity

The results of the antibacterial activity tests of various fractions and ethanol extract of cassava leaves on clinical isolates of *S. epidermidis* and *P. acnes* are shown in Table 5.

From Table 5, it could be seen that the water fraction of the ethanol extract of cassava leaves did not show antibacterial activity against clinical isolates of *S. epidermidis* or *P. acnes*. The n-hexane fraction showed only antibacterial activity at a concentration of 10% against *P. acnes* clinical isolates, whereas *S. epidermidis* clinical isolates showed no activity. Ethyl acetate fraction was the most active fraction because it had a larger inhibition zone both in clinical isolates of *S. epidermidis* and *P. acnes* compared with the other two fractions and ethanol extract as a comparison at a concentration of 10%. The selection of the lowest concentration for testing fraction activity was based on the antibacterial activity test of ethanol extract. However, in testing the antibacterial activity of extracts with a concentration of 5%, there was no activity against clinical isolates of *S. epidermidis* and its activity against clinical isolates of *P. acnes* was relatively small, so the second smallest concentration was used, i.e., 10%, as a concentration test to facilitate observation of test results fraction activity. The diameter of the inhibition zone was a parameter of a compound or substance that can still influence the bacteria. The greater diameter of the inhibition zone indicates that the bacteria were still sensitive to an antibacterial substance. And conversely, the smaller diameter of the inhibitory zone indicated that the bacteria began to be resistant to the antibacterial substances or compounds tested [38].

### 3.8. Determination of the MIC and MBC of the Fraction

The results of MIC and MBC determination of ethyl acetate fraction, as the most active fraction, against *S. epidermidis* and *P. acnes* clinical isolates using the microdilution method are shown in Table 6.

The MIC value was obtained from the smallest concentration which still inhibited bacterial growth, in the microplate, shown by the first turbid concentration While the MBC value was obtained from the smallest concentration that was able to kill almost all bacterial colonies, which was shown by the concentration of last clear microplate. Table 6 (culture subheading) showed that the MIC value of cassava leaf ethyl acetate fraction against bacterial isolates of *S. epidermidis* was in the concentration range of 1.25–2.5% and those of *P. acnes* clinical isolates in the concentration range of 0.625–1.25%. For MBC values of cassava leaf ethyl acetate fraction against bacterial isolates, *S. epidermidis* was at a concentration of 2.5% and clinical isolates of *P. acnes* at a concentration of 1.25%. However, the interpretation of these results of the data under culture in the above table was not valid because the determination of MIC and MBC was based on the results of visualization on the microplate well by referring to the position where a significant fraction of the pigment changes in the well from brown to cloudy white. Therefore, after testing microdilution, a subculture of the MIC and MBC fractions of ethyl acetate fraction was carried out on the test bacteria to ascertain which concentrations were MIC and MBC, and the results under subculture of Table 6 were obtained. From the determination of MIC and MBC ethyl acetate fraction on culture and subculture results, it could be concluded that the MIC value of cassava leaf ethyl acetate fraction against bacteria *S. epidermidis* is in the concentration range of 2.5%–5% and *P. acnes* clinical isolates were in the concentration range of 1.25%–2.5%. Whereas the MBC value of ethyl acetate fraction of cassava leaves to bacterial isolates of *S. epidermidis* was at a concentration of 5%, and the clinical isolates of *P. acnes* were at a concentration of 2.5%. The difference in interpretation of subculture data with the results on culture microplates was caused by turbidity of ethyl acetate fractions which still contained color pigments, so it was classified as undetectable (no bacterial growth) because the deposits at the base of the column in the MIC range during culture microplate observations could not be clearly seen.

### 3.9. Determination of the Most Active Thin-Layer Chromatography (TLC) Profile

**Figure 1:**
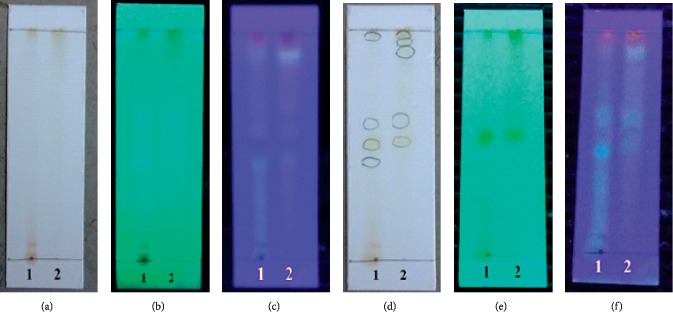
TLC profile of ethanol extract and ethyl acetate fraction of cassava leaves with the developer of n-butanol : acetic acid : water (4 : 1 : 5). (a) Visible light, (b) under UV 254 nm, (c) under UV 366 nm, (d) visible light + addition of AlCl_3_, (e) under UV 254 nm + addition of AlCl_3_, and (f) under UV 366 nm + addition of AlCl_3_. 1 indicates ethanol extract, and 2 indicates ethyl acetate fraction.

Determination of thin-layer chromatography (TLC) profile was carried out to determine the profile of the compounds contained in ethanol extract and the most active fraction of ethyl acetate from cassava leaves. From the results of the optimization that had been done, it resulted in the use of the stationary phase of silica gel GF 254 and the mobile phase in the form of an eluent n-butanol mixture : acetic acid : water (4 : 1 : 5). Observations were carried out under visible light, 254 nm UV light, and 366 nm UV light, and then with the addition of reagents, the appearance of AlCl3 spots. The results of determining the TLC profile of ethanol extract and the ethyl acetate fraction of cassava leaves are shown in Figure 1.

As shown in Figure 1, it is known that the ethanol extract of cassava leaves had four spots observed under a 366 nm UV lamp with the RF of 0.45 (light blue); 0.525 (dark blue); 0.6125 (dark blue); and 0.9875 (purplish red). Ethyl acetate fraction of cassava leaves had five observed spots under the 366 nm UV lamp with the RF of 0.525 each (dark blue); 0.625 (dark blue); 0.925 (light green); 0.9625 (light green); and 0.9875 (purplish red). After the addition of AlCl_3_ to spots on the TLC plate, there were changes in color spots observed under UV lamps of 254 nm and 366 nm, both in the spots of TLC extract of ethanol and in ethyl acetate fractions. This indicated that there was a reaction between the reagents of AlCl_3_ and the spot. The existence of color change reactions in spots might be due to the presence of flavonoid compounds that have free-hydroxyl groups and forms of complex bonds with the appearance of patches of AlCl_3_ on extracts and ethyl acetate fractions [39, 40]. Other observed spots had compounds from secondary metabolite groups identified in the phytochemical sample (polyphenol and quinone groups in the results of TLC extract and ethyl acetate fraction and saponin groups in the TLC extract results).

All data mentioned in this study can be used as a support that cassava leaves can be developed as an antiacne herbal ingredient. The use of herbs as antiacne has been proposed by other researchers [41–43].

## 4. Conclusions

From the results of this study, it concluded that the ethanol extract of cassava leaves (*M. esculenta* Crantz) had antibacterial activity against clinical isolates of *S. epidermidis* as well as on *P. acnes*. The most active fraction of ethanol extracted from cassava leaves was shown by ethyl acetate fraction. The MIC value of ethyl acetate fraction against *S. epidermidis* bacterial isolates at the concentration range of 2.5%–5% (w/v) and 1.25%–2.5% (w/v). The MBC value of ethyl acetate fraction against *S. epidermidis* clinical isolates was at a concentration of 5% (w/v), whereas clinical isolates of *P. acnes* was at a concentration of 2.5% (b/v). These results suggest that cassava leaves are possible to be developed into standardized antiacne herbal.

## Figures and Tables

**Table 1:** Phytochemical Screening of raw sample and ethanol extract of cassava leaves.

Secondary metabolite	Sample	Ethanol extract
Alkaloids	—	—
Flavonoids	+	+
Polyphenols	+	+
Tannin	—	—
Monoterpenoid and sesquiterpenoid	+	—
Steroids and triterpenoids	—	—
Quinone	+	+
Saponin	+	+

Description: + = detected; — = undetected.

**Table 2:** Standard sample and extract parameters.

Standard parameter	Concentrations (%)	
	Sample	Extract
Water content	9.2	10.0
The contents of the extract dissolved in water	16	60
The level of extract which was soluble in ethanol	27	74
Shrinkage drying	12	11
Total ash content	13	—
Levels of ash dissolved in water	92.31	—
Acid-insoluble ash level	7.69	—

**Table 3:** Biochemical test results of clinical isolates of *S. epidermidis*.

Biochemical test	Clinical isolate of *S. epidermidis*	*S. epidermidis* [34]
Motility	—	—
Glucose	+	+
Lactose	+	+
Mannose	+	+
Maltose	+	+
Saccharose	+	+
Triple sugar iron Agar (TSIA)	—	+
Urea	+	+
Methyl red (MR)	+	+
Voges–Proskauer (VP)	—	+
Citric	+	+

Notes: +: reacted; —: not reacted.

**Table 4:** Biochemical test results of clinical isolates of *P. acnes*.

Biochemical test	Clinical isolate of *P. acnes*	*P. acnes* [34, 36]
Motility	—	—
Glucose	—	+
Lactose	—	—
Mannose	—	—
Maltose	—	—
Saccharose	—	—
Triple sugar iron agar (TSIA)	+	+
Urea	+	+
Methyl red (MR)	+	+
Voges–Proskauer (VP)	—	—
Citric	+	+

Notes: +: reacted; —: not reacted.

**Table 5:** Antibacterial activity fractions and ethanol extract of. cassava leaves to *S. epidermidis* and *P. acnes* clinical isolates.

Test material	Inhibition zone (cm)		
	Concentration (%w/v)	Clinical isolate of *S. epidermidis*	Clinical isolate of *P. acnes*
The n-hexane fraction	10	—	1.46
Ethyl acetate fraction	10	1.46	1.97
Water fraction	10	—	—
Ethanol extract	10	1.27	**1.45**

The diameter of the capping hole = 0.9 cm.

**Table 6:** MIC and MBC ethyl acetate fraction of cassava leaves.

Concentration of ethyl acetate fraction (% w/v)	Bacterial growth			
	*S. epidermidis*		*P. acnes*	
	Clinical isolates		Clinical isolates	
	Culture	Subculture	Culture	Subculture
10	—	—	—	—
5	—	—	—	—
2.5	—	+	—	—
1.25	+	+	—	+
0.625	+	+	+	+
0.3125	+	+	+	+
0.15625	+	+	+	+
0.0781245	+	+	+	+
0.0390625	+	+	+	+

Description: — = no bacterial growth; + = bacterial growth.

## Data Availability

The MIC and the MBC data, as well as TLC data support this study.
